# Recurrence of preterm births: a population-based linkage with 3.5 million live births from the CIDACS Birth Cohort

**DOI:** 10.1002/ijgo.14053

**Published:** 2021-12-13

**Authors:** Aline S. Rocha, Rita de Cássia Ribeiro-Silva, Enny S. Paixao, Ila R. Falcão, Flavia Jôse O. Alves, Naiá Ortelan, Marcia F. de Almeida, Rosemeire L. Fiaccone, Laura C. Rodrigues, Maria Yury Ichihara, Mauricio L. Barreto

**Affiliations:** 1 School of Nutrition, Federal University of Bahia (UFBA), Salvador, Brazil; 2 Center for Data and Knowledge Integration for Health (CIDACS), Oswaldo Cruz Foundation, Salvador, Brazil; 3 Faculty of Epidemiology and Population Health, London School of Hygiene and Tropical Medicine; London, United Kingdom; 4 Institute of Collective Health, Federal University of Bahia, Salvador, Brazil; 5 School of Public Health, University of São Paulo (USP), São Paulo, Brazil; 6 Department of Statistics, Federal University of Bahia, Salvador, Brazil

**Keywords:** Birth Cohort, Poor populations, Preterm birth, Recurrent preterm birth

## Abstract

**Objective:**

To investigate the recurrence of preterm birth (PTB) among the poorest half of the Brazilian population.

**Methods:**

We conducted a population-based retrospective study in Brazil with the live births of multiparous women extracted from the CIDACS Birth Cohort between 2001 and 2015. We used multivariate logistic regression to estimate the odds of recurrent PTB in second and third births.

**Results:**

A total of 3,528,050 live births from 1,764,025 multiparous women were analyzed. The adjusted odds for the occurrence of a PTB given a previous PTB was 2.58 (95% CI 2.53-2.62). Lower gestational age increased the odds of a subsequent PTB [(aOR <28 weeks 3.61, 95% CI 3.41-3.83), (aOR 28 to 31 weeks 3.34, 95% CI 3.19-3.49), (aOR 32 to 36 weeks 2.42, 95% CI 2.38-2.47)]. Women who had two previous PTBs were at high risk of having a third (aOR 4,98 IC 95% 4.70-5.27). Recurrence of a PTB was more likely when the inter-birth interval was less than 12 months.

**Conclusions:**

In Brazil, a middle-income country, women with a previous PTB had an increased risk of a subsequent one. This association was affected by gestational age, the number of PTBs, severity of previous PTBs, and a short interval between births.

## Introduction

Preterm births (PTBs) account for more than 10% of all births worldwide, and subsequent complications are the leading cause of death in children under the age of five [1]. A preterm birth may have lifelong effects, including neurological and cognitive deficits, visual and hearing impairment, and an increased risk of chronic diseases in adulthood [2, 3]. Global estimates showed an increase in preterm birth rates, from 9.8% in 2000, to 10.6% in 2014, which is equivalent to an estimated 14.64 million preterm births out of 139.95 million live births [4]. In Brazil, 11.2% of births are premature, which places the country among the ten countries in the world with the highest PTB rates [4].

A previous PTB has been reportedly associated with a subsequent one. Genetic, environmental, and behavioral risk factors shared between two pregnancies may contribute to the recurrence of preterm birth through placental dysfunction, recurrent intrauterine infections, and other obstetric complications, such as diabetes and hypertension [5, 6]. A recent meta-analysis reported the absolute risk of PTB among women with a previous preterm birth to be 30% [7], in which the earlier the gestational age of the previous birth, the higher the risk of a subsequent preterm birth [8-10]. However, only studies from high-income countries were included in the meta-analysis, most were hospital-based, and had a limited sample size. Data from low and middle-income countries (LMICs) is in short supply. An example is a hospital-based study conducted in India with a sample of 291 women in which the PTB recurrence rate was estimated at 32% [11].

In Brazil, a previous PTB has been identified as an important risk factor for subsequent PTBs [12]. However, there are no studies estimating the magnitude of this association. Using data on more than 3.5 million live births from the CIDACS Birth Cohort, we investigated the recurrence of PTB among the poorest Brazilian population. A better understanding of the magnitude and effects of a previous PTB on a future pregnancy, especially among the disadvantaged population, is essential to assist policies and individual level care.

## Materials and methods

We conducted a population-based retrospective cohort study using the Centre for Data and Knowledge Integration for Health (CIDACS) Birth Cohort. This cohort was created by linking data from the national live birth system of Brazil (Sistema de Informação sobre Nascimentos - SINASC) and the 100 million Brazilian Cohort baseline for the period between January 1, 2001 and December 31, 2015.

The CIDACS Birth Cohort is composed of 24,695,617 live births. In general, the children included in the cohort were born from younger, unmarried, less educated mothers, and are more likely to be born via vaginal delivery, compared to children in the general Brazilian population[13]. In this study, we identified successive pregnancies using the unique maternal identifier and the newborn’s date of birth. This research was approved by the Federal University of Bahia Collective Health Institute (ISC-UFBA) research ethics committee (CAAE registration numbers: 41695415·0·0000·5030 and 18022319·4·0000·5030) and the London School of Hygiene and Tropical Medicine reference number 22817.

We obtained data from the Brazilian Live Births Information System (SINASC). SINASC includes information on the mother (e.g., maternal age, education level, marital status, and ethnicity); pregnancy information (e.g., antenatal appointments, length of gestation, and multiple fetuses); and information on the newborn (e.g., birth weight and sex) [14]. The 100 million Brazilian Cohort is primarily built from the Cadastro Único (CadUnico), a shared register for more than 20 social programs, which covers the poorest half of the Brazilian population (families with a monthly income equal to or below three minimum wages ~750 USD) [15].

We linked SINASC live birth records with the 100 million Brazilian Cohort using the following variables: mother’s name, maternal age at birth, maternal date of birth, and the mother’s municipality of residence at the time of delivery. Missing, implausible names, and duplicates were excluded. The linkage was performed using CIDACS RL-Record Linkage, a novel record-linkage tool developed to link large-scale administrative datasets at CIDACS [16, 17]. Linkage procedures were conducted at CIDACS in a strict data protection environment, and according to ethical and legal regulations [18].

**Figure 1:**
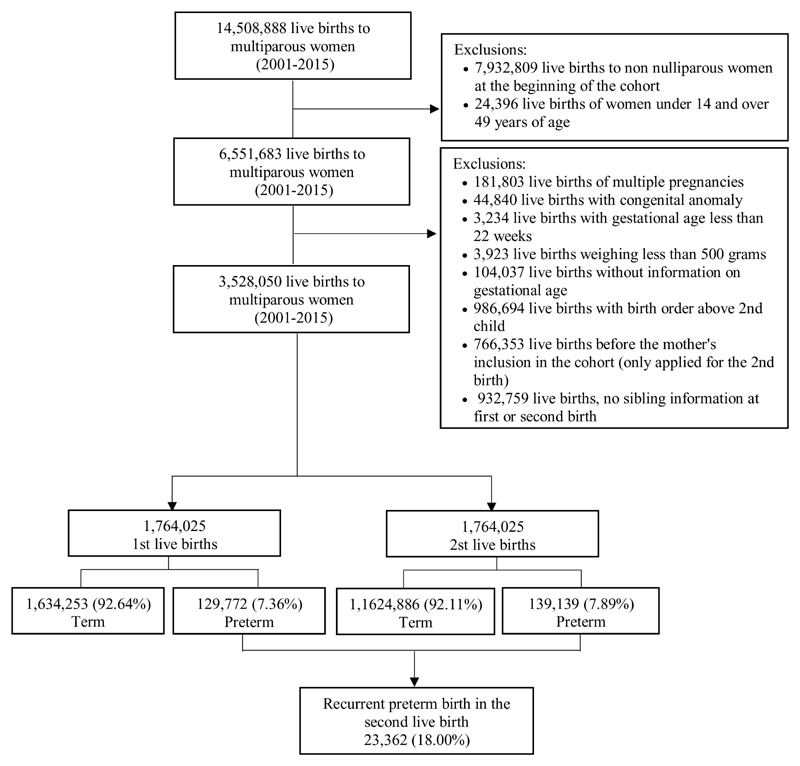
Study population flow diagram

The study population included live births of multiparous women aged between 14 and 49, who started to be followed up in the CIDACS Birth Cohort as nulliparous. We excluded all multiple births, live births with congenital anomalies, those weighing <500g, or with a gestational age < 22 weeks, and with information on gestational age missing. Also excluded were those with a birth date prior to the date of the mother’s entry into the cohort, and those with no sibling information (Figures 1 and S1).

Our main study outcome was PTBs in the second and third pregnancies, defined as a live birth at less than 37 weeks of gestation. Gestational age was defined as completed weeks. Since PTB tends to recur in a subsequent delivery, we compared offspring according to the gestational age at birth of the first pregnancy. Recurrence of preterm birth for the third birth was defined as a preterm birth after a preterm birth for the first and/or second pregnancy.

The following covariates were considered in the analyses: mother’s residential area (urban/rural), household overcrowding (≤2 inhabitants per room, or >2 inhabitants per room), mother’s self-declared race/skin color (white, mixed-race, black, or indigenous), mother’s level of education (≤3 years, 4-7 years, or ≥8 years of formal education), mother’s marital status (married: married, or in a stable relationship, or unmarried: single, divorced, or widowed), number of prenatal visits (none, 1 to 3 visits, 4 to 6 visits, or 7 or more visits), inter-birth interval (<12 months, 12 to 24 months, or ≥24 months), type of delivery (vaginal or cesarean) and maternal age (14-19 years old, 20-34 years old, or 35-49 years old). Household overcrowding, an important marker of poverty and social deprivation, [19] was calculated by dividing the number of individuals living in the house and the number of rooms. In the impossibility of estimating the inter-pregnancy interval, the inter-birth interval was estimated (in months) by the difference between the second or third child’s birth date and that of the previous child.

We employed logistic regression to calculate the odds ratio (OR) and 95% confidence intervals (95% CI) to estimate the association between preterm birth in the first pregnancy and the consequent risk in the second. The reference was the first pregnancy at term. We adjusted for mother’s residential area, family density, self-declared race/skin color, mother’s level of education, marital status, number of prenatal visits, maternal age, type of delivery, and newborn’s year of birth at the time of the first birth, to avoid introducing bias from factors that may have changed due to a poor outcome in the first birth. Individuals with missing observations in any of the variables were excluded from the multiple models.

We also estimated the odds ratio of a preterm birth in the third pregnancy using logistic regression, adjusted by the covariables mentioned above. The reference group was first and second pregnancy at term. The results of preterm birth were presented based on the order of birth to term and previous preterm births: Term / Term (reference category), Preterm / Term, Term / Preterm, and Preterm / Preterm.

Under the hypothesis that a short interval between pregnancies increases the chances of recurrent preterm births, we performed an analysis stratified by the inter-birth interval (<12 months, 12 to 24 months, and ≥24 months).

We performed additional analyses with live births after 2011 due to changes in the gestational age in the live birth records on SINASC from this date [20] (Supplementary Material).

All data was processed and analyzed using the STATA v15.1 program (Stata Corporation, 153 College Station, USA).

## Results

The CIDACS Birth Cohort population is composed of 14,508,888 live births from multiparous women. After applying the exclusion criteria, we selected 3,528,050 live births from 1,764,025 multiparous women to participate in this study. Overall, 129,772 (7.36%) of the women had a PTB in the first pregnancy and 139,139 (7.89%) in the second, 23,362 (18.00%) of which were classified as a recurrent preterm birth (Figure 1). The population characteristics according to the preterm birth status in the first pregnancy are described in Table 1. Compared to term live births, preterm live births were more likely among younger mothers who live in crowded households, and had attended fewer prenatal care appointments.

**Figure 2:**
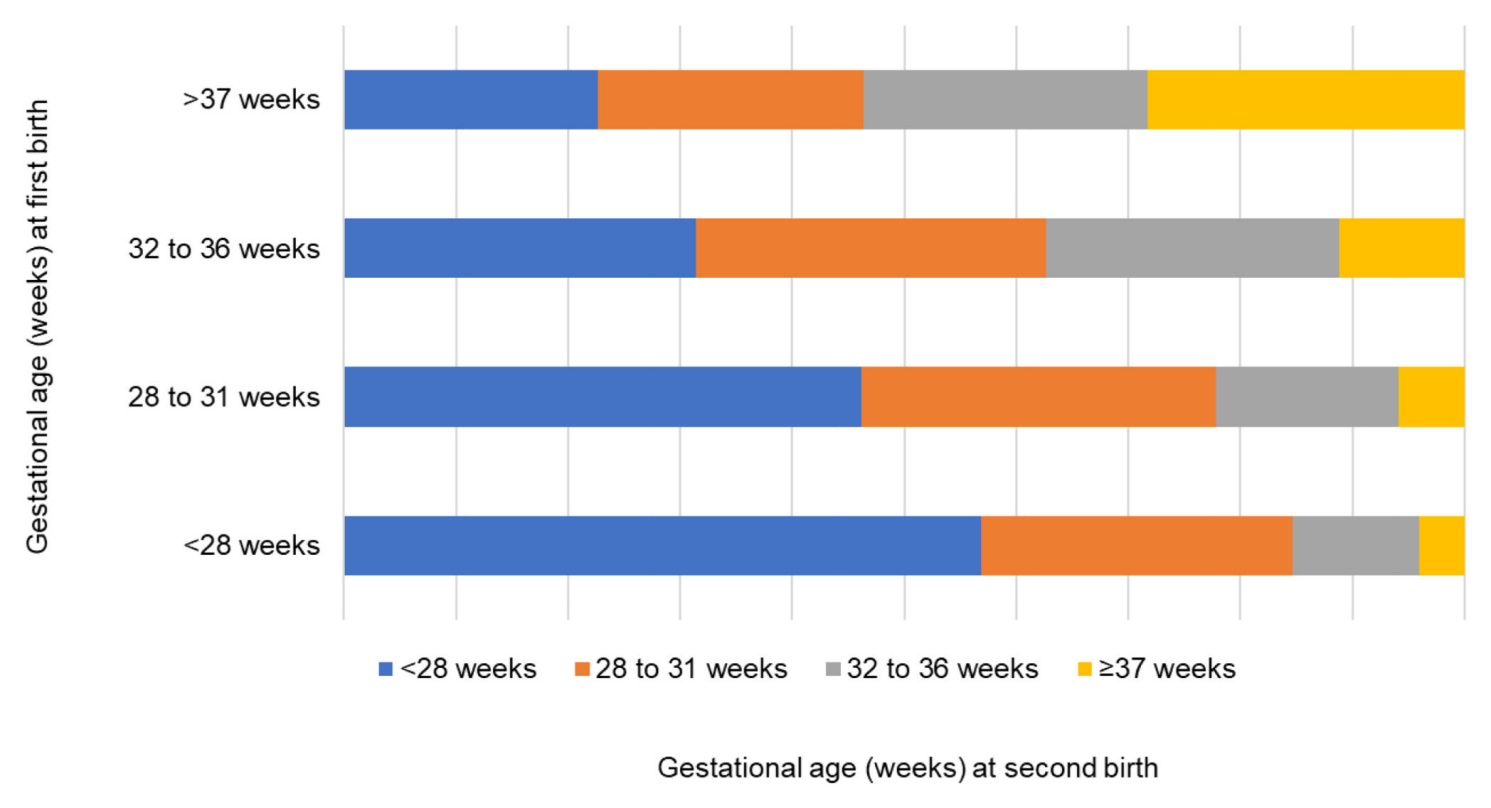
Gestational age at second birth by gestational age on the first birth.

**Figure 3:**
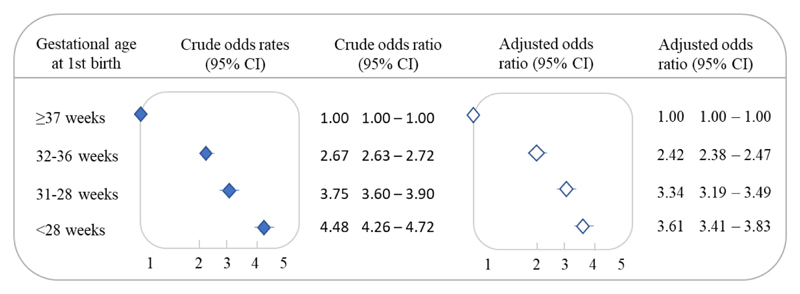
Recurrent preterm birth (<37 weeks of gestation) in the second pregnancy by gestational age of the first birth. 2001-2015 (n = 1,764,025). Unadjusted (filled diamonds) and adjusted (open diamonds) odds ratio by mother's residential area, household overcrowding, mother's self-declared race/skin color, mother’s level of education, mother's marital status, number of prenatal visits, maternal age, type of delivery, and newborn's year of birth at the time of the first birth.

The adjusted PTB odds ratio after a previous PTB was 2.58 (95% CI 2.53-2.62), compared to a first birth at term. We also observed that most of the second preterm births occurred in the same gestational age group as the first birth (Figure 2). Lower gestational age at the first birth increased the odds of a subsequent preterm birth, [(aOR _32 to 36 weeks_ 2.42, 95% CI 2.38-2.47), (aOR _28 to 31 weeks_ 3.34, 95% CI, 3.19-3.49), and (aOR _<28 weeks_ 3.61, 95% CI 3.41-3.83)] (Figure 3).

**Figure 4:**
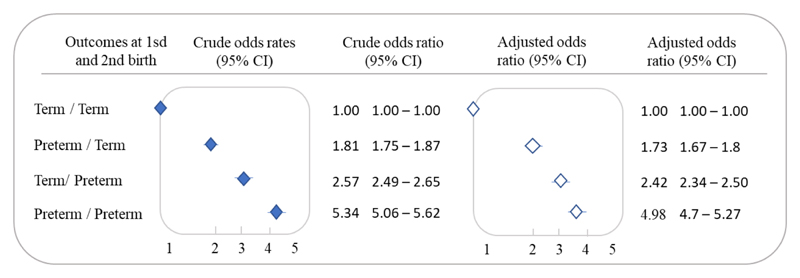
Recurrent preterm birth (<37 weeks) in the third pregnancy by term vs preterm birth in the first and second births, 2001-2015 (n = 544,665). Unadjusted (filled diamonds) and adjusted (open diamonds) odds ratio by mother’s residential area, household overcrowding, mother’s self-declared race/skin color, mother’s level of education, mother’s marital status, number of prenatal visits, maternal age, type of delivery, and newborn’s year of birth at the time of the first birth

Live births to women with preterm births in the two previous pregnancies (compared to those of women with two at term births) were 4.98 (95% CI 4.70-5.27) times more likely to result in a third preterm birth. The odds ratio of a third pregnancy with a premature delivery was 2.42 (95% CI 2.34-2.50) among live births of women with a first birth at term, followed by a preterm birth, and higher than among women with a preterm first birth and second birth at term 1.73 (95% CI 1.67-1.80) (Figure 4).

The analyses stratified by the inter-birth interval showed that the shorter the interval, the greater the risk of a recurrent preterm birth. The risk of recurrence was higher in the inter-birth interval of <12 months (aOR 2.86, 95% CI 2.60-3.15), followed by 12 to 24 months (aOR, 2.54, 95% CI 2.45-2.63), and ≥24 months (aOR 2.53; 95%CI 2.48-2.58) (Table 3). We also observed that the live births of women with two previous preterm births and an inter-birth interval of <12 months were more likely to have a third preterm birth (aOR 6.53, 95% CI 4.91-8.69), followed by 12 to 24 months (aOR 5.52, 95% CI 4.96-6.14), and ≥24 months (aOR 4.69, 95% CI 4.31-4.98) (Table 4).

An analysis restricted to births after 2011 had similar adjusted odds ratios (Tables S1 and S2).

## Discussion

In our study, live births to women with a previous PTB were over twice as likely to have a subsequent PTB, compared to those with a birth at term on their first pregnancy. In addition, the lower the gestational age at the first birth, the higher the odds of a subsequent preterm birth. The third birth of women with two previous preterm births was five times more likely to be a PTB, when compared to those with two previous at term births. For women with a history of one previous preterm and one previous term birth, the closer the last PTB, the higher the risk of a PTB in the third pregnancy.

This is the study with the largest sample size to estimate the risk of preterm birth in a second or third subsequent pregnancy using a population-based approach, and conducted in a middle-income country. Although the association between PTB in a previous and subsequent pregnancy had been observed, the mechanisms underlying this association are not well understood. It has been suggested that specific maternal factors can predispose women to premature births, since they have been associated with repeated placental complications, and are more susceptible to recurrent intrauterine infections and underlying disorders between pregnancies (for example, diabetes and hypertension) [5]. Similarly, risk factors shared between pregnancies (for example, smoking during pregnancy) may also contribute to the recurrence of a preterm birth [6, 21].

The risk of a subsequent preterm birth increased as the inter-birth interval decreased. The biological mechanisms that may explain this finding are related to the time it takes for the uterus to return to its normal state, including resolution of the inflammatory condition associated with the previous pregnancy [5]. A further explanation is the depletion of maternal vitamins and folate, since maternal stores of essential vitamins, minerals and amino acids are consumed during pregnancy, and a short interval decreases the opportunity to replace these nutrients between pregnancies [5, 22].

The results described in this study are consistent with literature [8, 9, 21, 23], except that the estimates of risk in these studies were much higher for the subsequent second or third birth. The reasons for these differences are unclear, but may be due to the differences in data sources, or the populations studied. One potential explanation for these differences may be that some studies included stillbirths in their analyses [8, 9]. The number of stillbirths that occur before 37 weeks is much higher than the number of preterm live births, which may increase the magnitude of the association. A further difference is our study population; we only included the poorest population from a middle-income country. Therefore, there is a more comprehensive array of structural and social causes associated with the occurrence of a preterm birth, which may have influenced the observation of the underlying biological probabilities estimated in this study. In all analyses, adjusting for demographic and obstetric factors reduced the estimated risk for recurrent preterm delivery, suggesting that unmeasured variables other than those observed, including the presence of chronic maternal diseases and infections, access to health care, and other social determinants (which are not available on our dataset), may play an important role in the occurrence of preterm births. The third point is the sample size, which was small and hospital-based in some studies, and this may have led to an overestimation of the measures, due to the inclusion of a higher proportion of high-risk pregnancies [21, 23]. Finally, this difference may have occurred due to the misclassification of preterm births in the Brazilian dataset. SINASC gathers secondary data on gestational age (GA) at birth. However, until 2010 the GA at birth was collected over wide intervals of gestational weeks [20], and the prematurity rate was considered underestimated, when compared to results from local studies with primary data collection [24]. From 2011, although SINASC started to collect the GA as a continuous variable, the mother’s last menstruation (LMP) was prioritized as a method of calculating the gestational age in weeks [20]. This can be a flawed method, due to circumstances such as individual variations in the menstrual cycle length, and recall biases in particular [24].

It is known that effective preventive measures and interventions during pregnancy can reduce the biological, social, and behavioral risk factors associated with preterm births [25]. Services provided during prenatal care for all pregnant women and women at high risk of preterm birth should include the identification and treatment of pre-existing conditions (e.g., diabetes, asthma, and other chronic conditions), sexually transmitted diseases, and other infections and pregnancy complications (e.g., hypertensive disorders of pregnancy, and antepartum hemorrhage); nutritional support, including multiple nutrient supplementation, and counseling to reduce risky behaviors, among others [25, 26]. There is increasing, almost universal coverage of prenatal care in Brazil. However, regional and social inequalities persist in the access to adequate prenatal care, contributing to the high premature birth rates observed in the country [27].

Our study has both strengths and weaknesses. This is the first study to assess the recurrence of PTB in a poor population of a middle-income country. The large sample size enabled the analysis of the recurrence of preterm birth in subsequent second and third births, and to perform an analysis stratified by the interval between births. However, this study has a number of limitations. Firstly, regarding the use of secondary data. The proportion of preterm births recorded on SINASC-Brazil was found to be underestimated by 15% [28], and misclassification, based on the criteria used to assess the gestational age at birth information (the date of the last menstrual period in most cases) may have occurred. However, they are probably non-differential errors and, therefore, our results may be underestimated, i.e., the magnitude of the association found may be even higher than that found in our analysis. Also, we were not able to classify the preterm birth subtypes (spontaneous, or with medical indication), due to a lack of information in our dataset. Secondly, residual confounding is possible, since data on maternal health conditions (e.g., co-morbidities such as diabetes and infections), as well as access, the quality of local health services, or special care for women with high-risk pregnancies, were not available in our dataset. Also, the database does not allow us to evaluate if cases with previous preterm births had any intervention in subsequent pregnancies. Thirdly, this study was conducted among the poorest population of a middle-income country with a history of major social and health inequalities, which may limit the generalizability of these findings.

In conclusion, our study showed an increased risk of a subsequent PTB in women who had a PTB in their previous pregnancy. This association was affected by gestational age, the number and order of previous preterm births, and the interval between births. These findings may contribute to clinical practice, the care of women with a history of previous preterm births, and to support policies for the prevention of high-risk pregnancies and preterm births. Our study highlights the importance of expanding access and the quality of prenatal care, introducing protocols for early identification, and the clinical management of women with a previous preterm birth, or who are at risk of a preterm birth, including a previous PTB birth, and applying timely therapeutic approaches. We recommend further research to analyze the impact of effective interventions in reducing PTB rates. Furthermore, studies are required in different low- and middle-income settings to uncover more evidence in such contexts, and for subsequent investigations according to PTB subtypes.

## Supplementary Material

Supplementary Figures and Tables

## Figures and Tables

**Table 1:** Mother's socio-demographic characteristics, prenatal care, and type of delivery on the first birth, 2001-2015 (n = 1,764,025)

First birth variables	Missing data	Total population		Term birth		Preterm birth	
		(n = 1,764,025)		(n = 1,634,253)		(n = 129, 772)	
	n° (%)	n°	%	n°	%	n°	%
**Urban/rural area of residence**							
Urban	72,395 (4.10)	1,303,734	77.07	1,205,132	76.88	98,602	79.46
Rural		387,896	22.93	362,403	23.12	25,493	20.54
**Household** overcrowding							
≤2 inhabitants per room	131,777 (7.47)	1,015,851	62.24	946,688	62.60	69,163	57.67
> 2 inhabitants per room		616,397	37.76	565,627	37.40	50,770	42.33
**Maternal race/ethnicity**							
White		541,650	33.45	501,731	33.44	39,919	33.55
Brown/Mixed race *“parda”*	144,507 (8.19)	934,694	57.71	866,655	57.76	68,039	57.17
Black		132,622	8.19	122,564	8.17	10,058	8.45
Indigenous		10,552	0.65	9,564	0.64	988	0.83
**Maternal education**							
≥ 8 years of formal study	26,192 (1.48)	951,910	54.78	881,011	54.72	70,899	55.48
4 to 7 years of formal study		654,220	37.65	605,495	37.61	48,725	38.13
≤3 years of formal study		131,703	7.58	123,538	7.67	8,165	6.39
**Marital status**							
Married, civil union	21,486 (1.22)	542,199	31.12	503,711	31.20	38,488	30.00
Single, divorced, widowed		1,200,340	68.88	1,110,540	68.80	89,800	70.00
**Number of prenatal visits**							
None		22,373	1.28	18,614	1.15	3,759	2.93
1 to 3 visits	16,542 (0.94)	143,507	8.21	119,919	7.41	23,588	18.41
4 to 6 visits		670,595	38.38	610,347	37.69	60,250	47.03
≥ 7 visits		911,006	52.13	870,501	53.76	40,505	31.62
**Maternal age at birth**							
14 to 19 years old	0 (0.0)	1,057,494	59.95	972,640	59.52	84,854	65.39
20 to 34 years old		697,675	39.55	653,556	39.99	44,119	34.00
35 to 49 years old		8,856	0.50	8,057	0.49	799	0.61
**Type of delivery**							
Vaginal	1,622 (0.09)	1,104,033	62.64	1,020,453	62.50	83,580	64.46
Cesarean		658,370	37.36	612,288	37.50	46,082	35.54

**Table 2:** Premature birth (<37 weeks of gestation) in the second live birth by preterm birth in the first live birth and inter-birth interval, 2001-2015 (n = 1,764,012)

**Inter-birth interval**	**Preterm on first birth**	**Preterm on second birth**			
		Unadjusted		Adjusted^a^	
		OR	(95% CI)	OR	(95% CI)
<12 months	≥37 weeks	Reference		Reference	
	<37 weeks	3·48	3·21 – 3.77	2.86	2.60 – 3.15
12 to 24 months	≥37 weeks	Reference		Reference	
	<37 weeks	2.97	2.88 – 3.06	2.54	2.45 – 2.63
≥24 months	≥37 weeks	Reference		Reference	
	< 37 weeks	2.70	2.65 – 2.75	2.53	2.48 – 2.58

a Analysis adjusted by mother's residential area, household overcrowding, mother's self-declared race/skin color, mother's level of education, mother's marital status, number of prenatal visits, maternal age, type of delivery, and newborn's year of birth at the time of the first birth.

**Table 3:** Recurrent preterm birth (<37 weeks) in the third live birth by term vs preterm birth in the first and second live births and inter-birth interval, 2001-2015 (n = 544,665)

**Inter-birth interval**	**First and second birth outcomes**	**Preterm on third birth**			
		Unadjusted		Adjusted^a^	
		OR	(95% CI)	OR	(95% CI)
<12 months	Term / Term	Reference		Reference	
	Preterm / Term	1.91	1.55 – 2.35	1.75	1.38 – 2.21
	Term / Preterm	3.36	2.88 – 3.93	3.15	2.65 – 3.74
	Preterm / Preterm	7.55	5.88 – 9.70	6.53	4.91 – 8.69
	Term /Term	Reference		Reference	
12 to 24 months	Preterm /Term	1.83	1.71 – 1.96	1.71	1.58 – 1.85
	Term / Preterm	2.77	2.62 – 2.93	2.59	2.43 – 2.76
	Preterm / Preterm	6.03	5.48 – 6.63	5.52	4.96 – 6.14
≥24 months	Term / Term	Reference		Reference	
	Preterm / Term	1.79	1.72 – 1.86	1.74	1.66 – 1.82
	Term / Preterm	2.37	2.27 – 2.46	2.28	2.18 – 2.38
	Preterm / Preterm	4.79	4.49 – 5.12	4.64	4.31 – 4.98

a Analysis adjusted by mother's residential area, household overcrowding, mother's self-declared race/skin color, mother's level of education, mother's marital status, number of prenatal visits, maternal age, type of delivery, and newborn's year of birth at the time of the first birth.

## Synopsis

In the poorest Brazilian population, the chance of preterm birth (PTB) was 2.58 times higher among women with a previous PTB, compared to a first birth at term.
