# Supplementary material for: Recurrence of preterm births: a population-based linkage with 3.5 million live births from the CIDACS Birth Cohort
Source: Int J Gynaecol Obstet. Author manuscript; Available in PMC 2022 Sep 1. (PMC7613286; doi:10.1002/ijgo.14053)
Supplement: Supplementary Figures and Tables [file EMS144934-supplement-Supplementary_Figures_and_Tables.docx]

#### **Supporting information**

14,508,888 live births to multiparous women

(2001-2015)

6,551,683 live births to multiparous women

(2001-2015)

2001-2015

- Exclusions:
- 7,932,809 live births to non nulliparous women at the beginning of the cohort
- 24,396 live births of women under 14 and over 49 years of age

Exclusions:

- 181,803 live births of multiple pregnancies
- 44,840 live births with congenital anomaly
- 3,234 live births with gestational age less than 22 weeks
- 3,923 live births weighing less than 500 grams
- 104,037 live births without information on gestational age
- 249,125 live births with birth order above 3rd child
- 148,150 live births before the mother's inclusion in the cohort (only applied for the 3rd birth)
- 4,182,556 live births, no sibling information at first, second or third birth

1,634,025 live births to multiparous women

(2001-2015)

544,675

3rd live births

544,675

2nd live births

544,675

1nd live births

40,012 (7.35%)

Preterm

504,663 (92.65%)

Term

506,528 (93.00%)

Term

38,147 (7.00%)

Preterm

46,595 (8.55%)

Preterm

498,080 (91.45%)

Term

**Recurrent preterm birth in the third live birth**

Preterm / Term 4,166 (12.63%)

Term / Preterm 5,295 (17.01%)

Preterm / Preterm 2,099 (29.89%)

**Figure S1**. Study population flow diagram

**Table S1:** Recurrent preterm birth (<37 weeks of gestation) in the second birth by gestational age of the first birth, 2011-2015 (n = 319,464)

| **Gestational age at first birth** | **Preterm on second birth** | | | |
| --- | --- | --- | --- | --- |
|  | Unadjusted | | Adjusted | |
|  | OR | (95% CI) | OR | (95% CI) |
| >37 weeks | Reference | | Reference | |
| 32 to 36 weeks | 2.19 | 2.12 – 2.26 | 1.99 | 1.93 – 2.06 |
| 31 to 28 weeks | 3.49 | 3.26 – 3.73 | 3.02 | 2.80 – 3.25 |
| < 28 weeks | 4.17 | 3.83 – 4.53 | 3.24 | 2.96 – 3.56 |

*Analysis adjusted by mother's residential area, household overcrowding, mother's self-declared race/skin color, mother's level of education, mother's marital status, number of prenatal visits, maternal age, type of delivery and newborn's year of birth.

**Table S2:** Recurrent preterm birth (<37 weeks) on the third live birth by term vs preterm birth on the first and second live births, 2011-2015 (n = 42,073)

| **First and second birth outcomes** | **Preterm on third birth** | | | | | | | |
| --- | --- | --- | --- | --- | --- | --- | --- | --- |
|  | Total population  (n = 42 073) | | Preterm birth  (n = 1191) | | Unadjusted | | Adjusted | |
|  | n | % | n | % | OR | (95% CI) | OR | (95% CI) |
| Term / Term | 32,170 | 76.46 | 3,096 | 9.62 | Reference | | Reference | |
| Preterm / Term | 3,599 | 8.55 | 531 | 14.75 | 1.63 | 1.47 – 1.78 | 1.51 | 1.35 – 1.69 |
| Term / Preterm | 4,946 | 11.76 | 930 | 18.80 | 2.17 | 2.01 – 2.36 | 2.10 | 1.92 – 2.29 |
| Preterm / Preterm | 1,358 | 3.23 | 453 | 33.38 | 4.70 | 4.17 – 5.29 | 4.41 | 3.87 – 5.03 |

*Analysis adjusted by mother's residential area, household overcrowding, mother's self-declared race/skin color, mother's level of education, mother's marital status, number of prenatal visits, maternal age, type of delivery and newborn's year of birth
